# The Expression of microRNAs and Their Involvement in Recurrent Pregnancy Loss

**DOI:** 10.3390/jcm13123361

**Published:** 2024-06-07

**Authors:** Maria-Markella Patronia, Anastasios Potiris, Despoina Mavrogianni, Eirini Drakaki, Theodoros Karampitsakos, Pavlos Machairoudias, Spyridon Topis, Athanasios Zikopoulos, Dionysios Vrachnis, Efthalia Moustakli, Chara Skentou, Ekaterini Domali, Nikolaos Vrachnis, Peter Drakakis, Sofoklis Stavros

**Affiliations:** 1 First Department of Obstetrics and Gynecology, Alexandra Hospital, Medical School, National and Kapodistrian University of Athens, 11528 Athens, Greece; mpatronia@med.uoa.gr (M.-M.P.); dmavrogianni@med.uoa.gr (D.M.); eirinidrak@med.uoa.gr (E.D.); 2 Third Department of Obstetrics and Gynecology, University General Hospital “ATTIKON”, Medical School, National and Kapodistrian University of Athens, 12462 Athens, Greece; apotiris@med.uoa.gr (A.P.); theokarampitsakos@hotmail.com (T.K.); pavlosmach@med.uoa.gr (P.M.); spyrostopis@med.uoa.gr (S.T.); thanzik92@gmail.com (A.Z.); dvrachnis@med.uoa.gr (D.V.); nvrachnis@med.uoa.gr (N.V.); pdrakakis@med.uoa.gr (P.D.); sfstavrou@med.uoa.gr (S.S.); 3 Laboratory of Medical Genetics, Faculty of Medicine, School of Health Sciences, University of Ioannina, 45110 Ioannina, Greece; thaleia.moustakli@gmail.com; 4 Department of Obstetrics and Gynecology, Medical School of the University of Ioannina, 45110 Ioannina, Greece; haraskentou@uoi.gr

**Keywords:** microRNAs (miRNAs), recurrent pregnancy loss, miscarriage, abortion, infertility

## Abstract

**Background:** Recurrent pregnancy loss refers to the spontaneous demise of two or more pregnancies before the 24 weeks of gestation. In almost half of the cases of recurrent miscarriages, the causes remain unknown since there is no reliable way of prognosis, early diagnosis, or treatment. Recent research has detected differential expression of certain miRNAs in reproductive system pathologies. **Methods:** The aim of the present review is to focus on microRNAs and their relationship with idiopathic recurrent miscarriages and to correlate miRNA expression with recurrent miscarriage and examine their potential role as biomarkers. Pubmed/Medline and Scopus databases were searched up to 31st January 2024 with terms related to recurrent pregnancy loss and miRNAs. **Results:** In total, 21 studies were selected for the review. A total of 75 different miRNAs were identified, showing a statistically significant differential expression. Around 40 miRNAs had increased expression, such as miR-520, miR-184 and miR-100-5p, 21 decreased, such as let-7c, and 14 had either increased or decreased expression depending on the study, such as miR-21. **Conclusions:** The dysregulation of miRNA expression is strongly associated with recurrent miscarriages. The circulating in the peripheral blood miRNAs, miR-100-5p and let-7c, might be utilized as biomarkers and establish a valuable non-invasive prognostic and diagnostic tool in the future.

## 1. Introduction

The term pregnancy loss (miscarriage) refers to the spontaneous demise of a pregnancy before the 24th week of gestation. According to the American Society of Reproductive Medicine and the European Society of Human Reproduction and Embryology, the term recurrent miscarriage refers to the loss of two or more pregnancies [1,2]. Recurrent miscarriages are a major complication of pregnancy, affecting 2–6% of couples trying to conceive [3]. The causes of recurrent miscarriage include endocrine dysfunctions, anatomic and environmental factors, antiphospholipid syndrome, thrombophilias, autoimmune diseases, genetic factors such as chromosomal abnormalities, and certain infections [4,5]. Genetic factors are the most common cause of recurrent miscarriages with an incidence of 30 to 50 percent [6]. However, in almost half of the cases of recurrent miscarriages, the causes remain unknown since there is no reliable way of prognosis, early diagnosis, or treatment [7].

MicroRNAs (miRNAs) are small non-coding RNA molecules approximately 22 nucleotides long, which regulate gene silencing by controlling the transcription of mRNAs into proteins. It is estimated that miRNAs control the expression of 60% of protein-coding genes in mammals, while more than 1000 miRNAs are produced in the human body, regulating at least one-third of all protein-coding genes [8]. miRNAs are involved in a multitude of biological processes, such as trophoblast development and differentiation, embryo activation and implantation, immune tolerance, and endometrial receptivity during implantation [9]. Therefore, the dysregulation of their expression may have serious consequences for these processes.

Recent research has detected differential expression of certain miRNAs in reproductive system pathologies. For instance, there are cases of women who experience recurrent implantation failure (RIF) in which increased expressions of miR-22, miR-145, and miR-31 were detected during the implantation window, compared to their levels in women with a history of uncomplicated pregnancies [10,11]. Moreover, normal embryo development depends on the proliferation, differentiation, and apoptosis of the trophoblast, which are processes regulated to a certain extent by miRNAs [12,13]. In the case of RPL, certain miRNAs show a modified expression pattern. Specific miRNAs, such as miR-184 and miR-100-5p have been found to be upregulated, while others, such as miR-126, show a significant downregulation in patients with RPL, compared to their levels in women with healthy pregnancies, who have never experienced RPL before. As there is growing interest in the action of miRNAs in RPL, the study of their expression is becoming increasingly common worldwide. Consequently, researchers focus on new screening biomarkers for early prenatal diagnosis. Hence, establishing a miRNA profile may be a key to deciphering miRNA activity and unveiling underlying pathogenic pathways linked to RPL.

In the present review, we focus on microRNAs and their relationship with idiopathic recurrent miscarriages, which present significant heterogeneity. The etiology of RPL is difficult to establish since multiple pathogenic mechanisms are involved. To the best of our knowledge, there are, currently, very few reviews focusing on the difference in miRNA expression and their relationship with RPL. This review correlates miRNA expression with recurrent miscarriage by citing the significantly dysregulated miRNAs that can be detected in the patient’s blood, the decidual and chorionic tissue of women with RPL, and matched controls. This may aid the development of a biomarker panel to be used for the prognosis and diagnosis of idiopathic recurrent miscarriages in the future.

## 2. Materials and Methods

In this systematic review, the published guidelines for Systematic Reviews and Metanalyses (PRISMA) were followed. The PRISMA checklist can be found in the Supplementary Materials. The search for scientific articles, regarding the expression of miRNAs in patients with recurrent miscarriages was carried out in the freely accessible PubMed/Medline search engines (https://pubmed.ncbi.nlm.nih.gov/, accessed on 1 February 2024) and Scopus (https://www.scopus.com/sources, accessed on 1 February 2024) up to 31st January 2024. The search query used included the following keywords: “miRNA*”, “microRNA*”, “miRNA expression”, “microRNA expression”, “recurrent pregnancy loss”, “recurrent miscarriage*”, and “recurrent abortion*”. These search terms were used in combination with the help of Boolean operators OR and AND. In each database, there was no time limit set or any other filter utilized.

The initial screening of the literature was performed independently by two authors (M-M.P. and A.P.). If a research paper was selected only by one author, the decision was made by a third author (D.M.). The inclusion criteria for the present review included studies about women with at least two consecutive miscarriages and a matched control group with at least one successful pregnancy or a history of legal termination of pregnancy without a history of miscarriage. In these groups, the expression of miRNAs should be investigated and compared among the two study groups with adequate primary data (number of subjects per population) and statistical measures for comparison (*p*-value or q-value and/or fold change). Similarly, the exclusion criteria included studies that (1) did not relate to miRNA expression or recurrent miscarriage, (2) did not provide sufficient data for data extraction, (3) included patients who were diagnosed with other causes of recurrent miscarriage such as reproductive tract infections, and (4) were written in another language than English.

Subsequently, for each included study, the following data were extracted: year of publication, country of origin, ethnic group of participated population, the diagnostic criteria for recurrent miscarriage (two or three consecutive miscarriages), the sample size (number of patients and controls), the mean age of patients and controls, the body mass index (BMI), the miRNAs investigated in each study, the miRNA detection methodology, and the tissue from which the miRNAs were isolated. The variable *p*-value or variable q-value, fold change variable, and/or miRNA relative expression variable were also extracted.

The protocol of the present systematic review was registered on the International Platform of Registered Systematic Review and Meta-Analysis Protocols (INPLASY) with registration number INPLASY202440116 and DOI identifier 10.37766/inplasy2024.4.0116 on the 29th of April 2024.

## 3. Results

### 3.1. Study Selection

**Figure 1:**
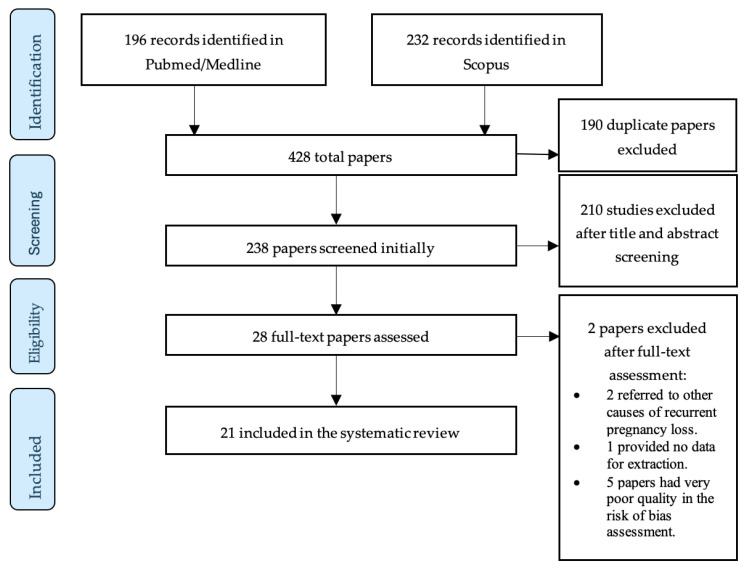
Flow diagram of study selection process.

In total, 21 studies were selected for the review. All included papers were published in the last decade. All included studies were case–control studies. Sixteen (76.19%) of the included studies use the occurrence of at least two consecutive miscarriages as the diagnostic criterion of RPL, while five (23.8%) use the occurrence of at least three consecutive miscarriages. The PRISMA flow diagram in Figure 1 schematically presents the stages of the article selection process.

### 3.2. Study Characteristics

In 14 (66.66%) studies, a blood sample was isolated and used for miRNA detection; in 6 (28.57%) studies, a chorionic villi sample was retrieved; and in 6 (28.57%) studies, a sample of the decidual tissue was examined. It is worth noting that in some studies more than one tissue was collected and tested for miRNA detection.

A total of 75 different miRNAs were identified, showing a statistically significant difference in expression between women with a history of recurrent miscarriage and the control group. Specifically, 40 (53.33%) miRNAs showed a significant increase in their expression, 21 (28%) miRNAs had a significantly decreased expression, and 14 (18.66%) miRNAs had both an increased and decreased expression in different studies. Table 1 summarizes the pooled results of all the included studies.

The control group is defined as pregnant women with at least one previous normal pregnancy and no history of RPL in 9 (42.85%) of the included studies and pregnant women with no history of RPL, but no recorded previous normal pregnancy in 12 (57.14%) of the included studies.

Regarding the tested tissue, 45 (60% of a total of 75 miRNAs) miRNAs from peripheral blood samples were found with a statistically significant differential expression in patients and controls. Among these miRNAs, 20 (26.66% of a total of 75 miRNAs) had an increased expression, 16 (21.33% of a total of 75 miRNAs) had a decreased expression, and 9 (12% of a total of 84 miRNAs) had both an increased expression in some studies and a decreased expression in some other studies. In addition, 23 (30.66% of a total of 75 miRNAs) miRNAs with a statistically significant differential expression were isolated from chorionic villus tissues. Additionally, 14 (18.66% of a total of 75 miRNAs) showed an increase in expression, and 9 (12% of a total of 75 miRNAs) showed a decrease in their expression. Finally, a total of 25 (33.33% of a total of 75 miRNAs) miRNAs were identified with a statistically significant difference in expression in the endometrium, of which 20 (26.66% of a total of 75 miRNAs) showed an increase in expression, 4 (5.33% of a total of 75 miRNAs) had a decreased expression, and 1 (1.33% of a total of 75 miRNAs) miRNA had both increased and decreased expression depending on the study. It should be noted that 14 (18.66%) out of 75 miRNAs were identified in more than one of the above-mentioned tissues. 

### 3.3. Risk of Bias of Included Studies

The NEWCASTLE–OTTAWA QUALITY ASSESSMENT SCALE for case–control studies [35] was used to assess the risk of bias in the selected studies. Among our studies, five provided no information on neither the selection of controls nor their comparability to the cases on the basis of the design or the analysis. As comparability is crucial and only addressed with a single item we decided not to take into consideration their findings. 

Regarding the remaining items no study provided information on response rates and the representativeness of the cases was not undoubted. Still, in all finally included studies, the cases and controls were well defined and comparable in all studies. The complete risk of bias assessment can be found in the Supplementary Materials.

## 4. Discussion

The present review depicts the important role of miRNAs in recurrent miscarriages. Our results show that the dysregulation of certain miRNAs in blood, and decidual and villus tissue is strongly associated with a high proportion of recurrent miscarriages and indicates their use as early prognostic and diagnostic biomarkers. The included studies highlight the participation of miRNAs in important signaling pathways related to the growth and differentiation of the trophoblast, the activation and implantation of the embryo, immune tolerance, and the receptivity of the endometrium. The dysregulation of their expression appears to have serious consequences in these processes, causing miscarriages. The key findings of this systematic review are summarized in Table 2.

MiRNAs regulate gene expression by controlling the transcription of mRNAs in proteins. Once miRNAs are produced, they bind in an mRNA, according to the base-pairing rule, and degrade it or repress its transcription. Recent studies highlight the role of certain miRNAs in the molecular mechanisms of RPL. MiR-184 and miR-520, for instance, seem to promote apoptosis and repress the proliferation of trophoblast cells by targeting WIG1 and PARP1, respectively [22,36]. Therefore, when overexpressed, they induce early spontaneous abortion. On the other hand, immune-regulatory miR-155-5p, which is downregulated in RPL patients, exhibits anti-inflammatory effects on human decidua stromal cells by regulating the NF-kB pathway signaling [21]. In addition, miR-126 enhances the expression of VEGF, an essential factor in fetal and placental angiogenic development. The downregulation of miR-126 may result in abnormalities in placental vasculature and cause spontaneous abortion [39]. 

Zhang et al. demonstrated that the overexpression of miR-155-5p reduces the secretion of inflammatory cytokines in endometrial cells promoting growth and proliferation of these cells. On the other hand, the reduction of miR-155-5p expression in women with a history of miscarriage leads to the apoptosis of endometrial cells through NF-κB pathway signaling [21]. The expression of miR-155 was also studied by Yan et al. in T-cells, as well as by Li et al. in NK cells of the endometrium of women with a history of recurrent miscarriages. The results of the two studies are aligned on increased expression of miR-155 in women who experienced miscarriages, and the authors suggest the possibility of using this miRNA as a possible future biomarker for recurrent miscarriage prognosis and treatment strategies [14,26].

miR-21 and members of the let-7 family are well-studied miRNAs regarding implantation and assisted reproduction. The overexpression of miR-21 leads to an increase in fertilized oocytes and embryos that reach the blastocyst stage. A decreased expression of miR-21 has the exact opposite results [40]. In addition, there is a differential expression of miRNAs between dormant and activated blastocysts. Five members of the let-7 family, most notably let-7a, appear to modulate the implantation potential of the activated blastocyst. In mouse models, the introduction of estradiol reduces the expression of let-7a, leading the dormant blastocyst to become activated [41]. 

Overexpression of miR-184 has been found in women with recurrent miscarriages. miR-184 targets specific genes in trophoblast cells leading to the apoptosis of trophoblast cells and consequently miscarriages [22]. Thus, the authors indicated the pivotal role of miR-184 in maintaining pregnancy and the possibility to be used as a diagnostic and therapeutic indicator in recurrent miscarriages. The same results were also supported by Jairajpuri et al., where authors associated the increased expression of miR-184 with an increased risk for recurrent pregnancy loss [29,36].

miR-520 has been found to be overexpressed in miscarriages. The overexpression of miR-520 has been also implicated in causing multiple DNA damages in trophoblast cells, leading to their apoptosis and embryo abortion [23]. Other studies have shown that the overexpression of miR-125b-1-3p and miR-29b reduces the ability of trophoblast infiltration during implantation through the disruption of vascular stability [42,43]. On the contrary, Tian et al. reported similar effects on trophoblast apoptosis and spontaneous abortions through the overexpression and synergistic action of miR-494 and miR-19b. Thus, the authors propose that this pair of mRNAs can be useful in the prediction and treatment of recurrent abortions [30].

The results on the effect of miR-146a in RPL are conflicting in the literature. Wang et al., studying the differential expression of miR-146a in chorionic villi and endometrial tissues of women with recurrent miscarriages, found increased expression and supported its participation in signaling pathways of recurrent miscarriages [15]. Tian et al. presented similar results suggesting the involvement of miR-146a in the abnormal function of trophoblast cells in recurrent miscarriages [30]. However, Yan et al. reported decreased expression of miR-146a in women with recurrent miscarriages. Moreover, the authors suggested that the decreased expression of miR-146a can be used as a diagnostic biomarker of miscarriage [26]. The need for further studies of miR-146a and its association with miscarriages should be highlighted.

The use of circulating miRNAs in the blood as biomarkers of recurrent miscarriages was also supported by Yang et al. Among all the miRNAs, the researchers focused on increased expression of miR-100-5p in the blood of women with recurrent miscarriages [28]. This finding is supported by the results of other studies too; hence, the authors propose that the expression of miR-100-5p might be useful not only as a diagnostic and prognostic biomarker for recurrent miscarriages but also for predicting the outcome of IVF attempts [15,20,31]. 

Focusing on miRNA expression, target genes, and the related mechanisms may contribute to a better understanding of the unknown causes of recurrent pregnancy loss. The present review highlights the miRNAs with statistically significant differential expression, proposing the establishment of a direct association between miRNA expression and RPL. It should be noted that although most of the above studies show statistically significant changes in miRNA expression in women with RPL, better standardization of methodology is required to improve the subsequent analyses. Additionally including large cohorts of women with miscarriages and uncomplicated pregnancies (controls) may strengthen the statistical power of the results. To summarize, the use of miRNA expression in women with recurrent miscarriages has prospects for finding new non-invasive prognostic and diagnostic biomarkers. The presented findings may also improve personalized therapeutic strategies in the future.

## 5. Conclusions

In conclusion, the dysregulation of miRNA expression is strongly associated with a high percentage of recurrent miscarriages. A large number of studies highlight their differential expression as a potential future biomarker of recurrent pregnancy loss. However, the data are conflicting in some cases. The greatest prospects are the miRNAs circulating in the peripheral blood, such as miR-100-5p and let-7c, since their expression in this tissue has been extensively studied. These miRNAs might be utilized as biomarkers and can be established as a valuable non-invasive prognostic and diagnostic tool in the future. Τhe need for further research in the field of miRNA expression and their use for diagnostic purposes in recurrent miscarriages should highlighted.

## Figures and Tables

**Table 1:** Pooled results of included studies.

Study	Country of Publication	Cases (N)	Controls (N)	Case’s Age (Years)	Case’s BMI (kg/m²)	Tissue	miRNA	miRNA Expression (Cases vs. Controls)	Fold Change	*p*-Value
Li, 2016 [14]	China	20	20	31.0 ± 2.87		Decidual tissue	miR-34a	Upregulated		<0.001
							miR-155	Upregulated		<0.001
							miR-141	Upregulated		<0.01
							miR-125a	Upregulated		<0.001
							miR-125b	Upregulated		<0.001
							miR-24	Downregulated		<0.01
Wang J., 2016 [15]	China	18	15	29.61 ± 4.41		Decidual tissue	miR-516a-5p	Upregulated	11.0194	
							miR-517a-3p	Upregulated	10.9882	
							miR-519a-3p	Upregulated	27.4134	
							miR-519d	Upregulated	8.104	
							miR-520a-3p	Upregulated	10.755	
							miR-520h	Upregulated	15.4828	
							miR-455-5p	Upregulated	2.1923	
							miR-1323	Upregulated	6.7395	
							miR-516b-5p	Upregulated	8.6609	
							miR-205-5p	Upregulated	9.0955	
							miR-522-3p	Upregulated	21.7944	
						Villus tissue	miR-1	Downregulated	0.2173	
							miR-372	Downregulated	0.228	
							miR-100-5p	Upregulated	3.7043	
							miR-24-3p	Upregulated	2.8975	
							miR-146a-5p	Upregulated	4.4543	
							miR-371a-5p	Downregulated	0.2574	
							miR-486-5p	Downregulated	0.3089	
							miR-376c-3p	Downregulated	0.2874	
							miR-191-5p	Upregulated	2.1158	
Tutunfroush M., 2021 [16]	Iran	60	60	34.60 ± 3.96		Blood	miR-23a-3p	Downregulated	4.66	0.053
							miR-101-3p	Downregulated	12.48	0.03
							miR-let-7c	Downregulated	11.21	0.007
Geng X., 2022 [17]	China	36	40	29.1 ± 3.4		Villus tissue	miR-33a	Downregulated		0.0021
							miR-33b	Downregulated		0.0043
							miR-181a	Downregulated		<0.05
							miR-92a	125		
							miR-155	No significant change		
							miR-148a	No significant change		
Abbaskhani H., 2022 [18]	Iran	50	50	<35		Blood	miR-361-3p	Downregulated	1.61	<0.0001
Manzoor U., 2022 [19]	India	40				Blood/ Plasma	miR-125a	Downregulated		0.0001
Xu N., 2022 [20]	China	20	21	26.41	20.3	Blood/ Plasma	miR-520a-3p		1.48 × 10^−7^	6.57 × 10^−9^
							miR-100-5p		12.4101	0.02878817
							miR-101-3p		0.7062	0.67274972
							miR-127-3p		76.3589	0.01062173
							miR-146a-5p		2.0031	0.46514969
							miR-146b-5p		1.1814	0.83534061
							miR-155-5p		223.627	0.00259653
							miR-486-5p		0.872	0.8601277
							miR-24-3p		1.6082	0.52987702
							miR-23a-3p		95.4484	0.00121073
							miR-361-3p		29.815	0.0037326
							miR-320b		0.4011	0.35605988
							miR-455-5p		25.013	0.06849887
							miR-1323		1.2705	0.92631658
							miR-516b-5p		1.9772	0.603201
							miR-205-5p		370.6108	0.00940756
							miR-376c-3p		10.0797	0.34838009
							miR-191-5p		1.4426	0.65767043
							miR-92a		0.7423	0.69359217
							miR-148b-3p		0.4685	0.31694608
							miR-221-3p		1177.7001	3.83 × 10^−5^
							miR-30d-5p		0.7484	0.70561117
							miR-99b-5p		3.3575	0.17988086
							miR-23b-3p		656.5436	3.17 × 10^−5^
							miR-204-3p		14.2344	0.26530357
							miR-22-5p		0.7652	0.88520907
							miR-206		512.4195	0.000782531
							miR-214-3p		8566539.006	6.75 × 10^−10^
							miR-184		0.872	0.945970165
Zhang Q., 2021 [21]	China	30	40			Decidual tissue	miR-155-5p	Downregulated		<0.01
						Blood/Serum		Downregulated		<0.01
Zhang Y., 2019 [22]	China	9	9	28.37 ± 1.46		Decidual tissue/Stromal cells	miR-184	Upregulated		<0.05
		9	9	28.78 ± 2.39		Decidual tissue/Immune cells		Upregulated		<0.0001
		54	14	32.31 ± 1.04		Blood		Upregulated		<0.05
Dong X., 2017 [23]	China	11	13	25.30 ± 2.39		Villus tissue	miR-520	Upregulated		<0.05
Zhao W., 2017 [24]	China	40	40	28.8		Decidual tissue	miR-365	Upregulated		<0.001
		14	13				miR-150	Upregulated		<0.01
							miR-10a	Upregulated		<0.01
							miR-27c	Upregulated		<0.001
							miR-30c	Downregulated		<0.001
							miR-20	Downregulated		<0.01
							miR-24	Downregulated		<0.01
							miR-181	Downregulated		<0.01
Qin W., 2016 [25]	China	27	28	29.10 ± 4.22		Blood/ Plasma	miR-101-3p	Upregulated	3.614	0.01932
							miR-320b	Upregulated	2.637	0.00218
							miR-146b-5p	Upregulated	5.108	0.00869
							miR-92a	Upregulated	2.662	0.03084
							miR-148b-3p	Upregulated	4.595	0.04343
							miR-221-3p	Upregulated	7.409	0.00704
							miR-30d-5p	Upregulated	4.361	0.02854
							miR-99b-5p	Upregulated	9.743	0.00842
							miR-23b-3p	Upregulated	3.43	0.021
							miR-204-3p	Downregulated	0.394	0.03839
							miR-22-5p	Downregulated	0.337	0.01717
							miR-559	Downregulated	0.39	0.01504
Yan Y., 2023 [26]	China	50	50	27.28 ± 3.10	19.87 ± 2.38	Blood	miR-106b	Upregulated		<0.05
							miR-93	Upregulated		<0.05
							miR-25	Upregulated		<0.05
							miR-146a	Downregulated		<0.05
							miR-155	Downregulated		<0.05
							miR-326	No significant change		
Hosseini Μ.Κ., 2018 [27]	Turkey	16	8	30.23 ± 5.81		Blood/ Plasma	miR-let-7c	Downregulated	−2.68	0.0323
							miR-122	Upregulated	1.57	0.0241
							miR-135a	Upregulated	3.8	0.0249
						Villus tissue	miR-125a-3p	Upregulated	1.72	0.0258
							miR-3663-3p	Upregulated	2.02	0.0075
							miR-423-5p	No result	No result	No result
							miR-575	Upregulated	2.5	0.00029
Yang Q., 2018 [28]	China	16	29	29.9 ± 0.84		Villus tissue	miR-23a-3p	No significant change		
							miR-27a-3p	Upregulated		<0.05
							miR-29a-3p	Upregulated		<0.05
							miR-100-5p	Upregulated		<0.01
							miR-127-3p	No significant change		
							miR-486-5p	No significant change		
						Blood/ Plasma	miR-23a-3p	No significant change		
							miR-27a-3p	Upregulated		<0.05
							miR-29a-3p	Upregulated		<0.01
							miR-100-5p	Upregulated		<0.01
							miR-127-3p	Upregulated		<0.05
							miR-486-5p	Downregulated		<0.05
						Blood/Serum	miR-23a-3p	Downregulated		<0.05
							miR-27a-3p	No significant change		
							miR-29a-3p	No significant change		
							miR-100-5p	No significant change		
							miR-127-3p	Downregulated		<0.05
							miR-486-5p	Upregulated		<0.05
Jairajpuri D.S., 2021 [29]	Bahrain	20	20	30.4 ± 3.6	23.6 ± 4.2	Blood/ Plasma	miR-let-7e	Upregulated	12.144	0.0019
							miR-221-3p	Upregulated	9.846	0.0041
							miR-16	Upregulated	9.122	0.0024
							miR-519d	Upregulated	7.223	0.0044
							miR-184	Upregulated	6.816	0.0013
							miR-410	Upregulated	5.514	0.0027
							miR-21	Downregulated	0.347	0.0061
							miR-125	Downregulated	0.301	0.0051
							miR-let-7a	Downregulated	0.144	0.0007
							miR-let-7d	Downregulated	0.121	0.0084
Tian S., 2020 [30]	China	19	16	32.26 ± 0.97		Villus tissue	miR-19b	Downregulated	2.19	0.016
							miR-494	Upregulated	2.85	0.021
							miR-146a	Upregulated	2.33	0.0199
							miR-133a	Upregulated	1.08	0.8526
							miR-21	Upregulated	2.07	0.0352
							miR-125b	Upregulated	1.76	0.1138
							miR-196a	Upregulated	2.17	0.056
							miR-149	Upregulated	2.06	0.059
							miR-17	Upregulated	1.53	0.23
Bruno V., 2022 [31]	Italy	20 untreated cases	18	32.7 ± 4.9	24.49 ± 5.5	Blood	miR-184	Upregulated	79.57	0.000003
							miR-205-5p	Upregulated	5.74	0.015287
							miR-100-5p	Upregulated	1.87	0.002505
							miR-34a	Upregulated	1.7	0.036288
							miR-376c-3p	Downregulated	−2.07	0.000007
							miR-29a-3p	Downregulated	−2.34	0.004481
							miR-155-5p	Downregulated	−3.67	0.008714
							miR-23a-3p	Downregulated	−4.47	0.000003
							miR-30d-5p	Downregulated	−6.13	0.000692
							miR-27a-3p	Downregulated	−8.32	0.000001
							miR-146a-5p	Downregulated	−11.31	0.000015
							miR-191-5p	Downregulated	−14.46	0.000125
							miR-24-3p	Downregulated	−35.57	0.000001
							miR-206	Downregulated	158.67	0.000002
							miR-92a-3p	Upregulated	1.8	0.000317
							miR-221-3p	Downregulated	−201.16	0.000001
		18 treated cases	18	34.8 ± 4.4	22.45 ± 3.9		miR-29a-3p	Downregulated	3.61	0.000004
							miR-23a-3p	Downregulated	−3.29	0.000001
							miR-24-3p	Upregulated	1.82	0.000013
							miR-214-3p	Upregulated	3.93	0.000049
Zhao L., 2018 [32]	China	29	35	33.3 ± 5.2		Decidual tissue	miR-146a-5p	Downregulated		0.027
Parhizkar F., 2023 [33]	Iran	40	40	31.2 ± 4.48	28.19 ± 3.92	Blood	miR-206	Upregulated		0.0038
							miR-30a	Upregulated		0.0123
							miR-18a	Downregulated		0.0101
Al-Rubaye S., 2021 [34]	Iran	50	50	<35		Blood	miR-214-3p	Upregulated		<0.01

**Table 2:** Key findings of the systematic review.

miRNA	Tissue	Regulation	References
miR-184	Decidual/Blood	Up	[20,22,29,31,36]
miR-520	Villus/Decidual	Up	[15,20,23,36]
miR-155/-5p	Villus/Blood	Up, Down/Up	[14,17,20,21,26,31,37]
miR-100-5p	Decidual/Blood	Up	[15,20,28,31]
miR-146a/-5p	Villus/Decidual/Blood	Down/Up/Down	[15,20,26,30,31,32]
let-7c	Blood	Down	[16,27]
miR-21	Decidual/Blood	Up/Down	[29,30,37,38]
miR-23a-3p	Blood	Down	[16,20,28,31]
miR-92a/-3p	Blood	Up	[17,20,25,31]
miR-221-3p	Blood	Up/Down	[20,25,29,31]

## Data Availability

Not applicable.

## Supplementary Materials

The following supporting information can be downloaded at: https://www.mdpi.com/article/10.3390/jcm13123361/s1, Table S1: Prisma Checklist; Table S2: Risk of Bias Assessment; Table S3: Search Strategy.
