# Supplementary material for: The Expression of microRNAs and Their Involvement in Recurrent Pregnancy Loss
Source: J Clin Med. 2024 Jun 7;13(12):3361. doi: 10.3390/jcm13123361 (PMC11203554; doi:10.3390/jcm13123361)
Supplement: Supplementary file 1 [file jcm-13-03361-s001.zip › jcm-2999257-supplementary/Patronia & Potiris - Risk of Bias Assesment.pdf]

	Selection				Comparability	Exposure		
ID	Is the case definition adequate?	Representativeness of the cases	Selection of Controls	Definition of controls	Comparability of cases and controls on the basis of the design or analysis	Ascertainment of exposure	Same method of ascertainment for cases and controls	Non-Response rate
Li, 2016 [14]	*		*	*	*	*	*	
Wang J., 2016 [15]	*			*	**	*	*	
Tutunfroush M., 2021 [16]	*			*	*	*	*	
Geng X., 2022 [17]	*		*	*	*	*	*	
Abbaskhani H., 2022 [18]	*		*	*	*	*	*	
Manzoor U., 2022 [19]	*	*	*	*	**	*	*	
Xu N., 2022 [20]	*	*	*	*	*	*	*	
Zhang Q., 2021 [21]	*		*	*		*	*	
Zhang Y., 2019 [22]	*		*	*		*	*	
Dong X., 2017 [23]	*			*	*	*	*	
Zhao W.,2017 [24]	*	*	*	*	*	*	*	
Qin W.,2016 [25]	*		*	*	*	*	*	
Yan Y., 2023 [26]	*	*	*	*	*	*	*	
Hosseini M.K., 2018 [27]	*			*	*	*	*	
Yang Q., 2018 [28]	*			*	*	*	*	
Jairajpuri D.S., 2021 [29]	*			*	*	*	*	
Tian S., 2020 [30]	*			*	*	*	*	
Bruno V., 2022 [31]	*	*	*	*	*	*	*	
Zhao L., 2018 [32]	*	*	*	*	*	*	*	
Parhizkar F., 2023 [33]	*		*	*	*	*	*	
Al-Rubaye S., 2021 [34]	*		*	*	*	*	*	
