# Supplementary material for: The Expression of microRNAs and Their Involvement in Recurrent Pregnancy Loss
Source: J Clin Med. 2024 Jun 7;13(12):3361. doi: 10.3390/jcm13123361 (PMC11203554; doi:10.3390/jcm13123361)
Supplement: Supplementary file 1 [file jcm-13-03361-s001.zip › jcm-2999257-supplementary/Patronia & Potiris - Search Strategy.pdf]

The expression of microRNAs and their involvement in recurrent pregnancy loss

Search strategy

Group A	Group B
miRNA*	recurrent pregnancy loss
microRNA*	recurrent miscarriage*
miRNA expression	recurrent abortion*
microRNA expression	

Search strategy: (All Terms of Group A with an OR administrator) AND (All Terms of Group B with an OR administrator)

PubMed/Medline Search Query

((((miRNA*) OR (microRNA*)) OR (miRNA expression)) OR (microRNA expression)) AND (((recurrent pregnancy loss) OR (recurrent miscarriage*)) OR (recurrent abortion*))

Scopus Search Query

(TITLE-ABS-KEY (mirna*) OR TITLE-ABS-KEY (microrna*) OR TITLE-ABS-KEY (mirna AND expression) OR TITLE-ABS-KEY (microrna AND expression)) AND (TITLE-ABS-KEY (recurrent AND pregnancy AND loss) OR TITLE-ABS-KEY (recurrent AND miscarriage*) OR TITLE-ABS-KEY (recurrent AND abortion*))
